# Effects of rehabilitative interventions on pain, function and physical impairments in people with hand osteoarthritis: a systematic review

**DOI:** 10.1186/ar3254

**Published:** 2011-02-18

**Authors:** Liuzhen Ye, Leonid Kalichman, Alicia Spittle, Fiona Dobson, Kim Bennell

**Affiliations:** 1 Rehabilitative Services Department, Changi General Hospital, 2 Simei Street 3, 529889 Singapore; 2 Department of Physiotherapy, School of Health Sciences, The University of Melbourne, 200 Berkeley Street, Victoria, 3010, Australia; 3 Department of Physical Therapy, Recanati School for Community Health Professions, Faculty of Health Sciences, Ben-Gurion University of the Negev, PO Box 653, Beer Sheva, 84105, Israel; 4 Victorian Infant Brain Studies, Murdoch Childrens Research Institute, Flemington Road, Parkville, 3052, Australia; 5 Newborn Research, Royal Women's Hospital, Melbourne, Grattan Street & Flemington Road, Parkville, 3052, Australia; 6 Centre for Health, Exercise and Sports Medicine, Department of Physiotherapy, School of Health Sciences, The University of Melbourne, 200 Berkeley Street, Victoria, 3010, Australia; 7 Murdoch Childrens Research Institute, Flemington Road, Parkville, 3052, Australia

## Abstract

**Introduction:**

Hand osteoarthritis (OA) is associated with pain, reduced grip strength, loss of range of motion and joint stiffness leading to impaired hand function and difficulty with daily activities. The effectiveness of different rehabilitation interventions on specific treatment goals has not yet been fully explored. The objective of this systematic review is to provide evidence based knowledge on the treatment effects of different rehabilitation interventions for specific treatment goals for hand OA.

**Methods:**

A computerized literature search of Medline, the Cumulative Index to Nursing and Allied Health Literature (CINAHL), ISI Web of Science, the Physiotherapy Evidence Database (PEDro) and SCOPUS was performed. Studies that had an evidence level of 2b or higher and that compared a rehabilitation intervention with a control group and assessed at least one of the following outcome measures - pain, physical hand function or other measures of hand impairment - were included. The eligibility and methodological quality of trials were systematically assessed by two independent reviewers using the PEDro scale. Treatment effects were calculated using standardized mean difference and 95% confidence intervals.

**Results:**

Ten studies, of which six were of higher quality (PEDro score >6), were included. The rehabilitation techniques reviewed included three studies on exercise, two studies each on laser and heat, and one study each on splints, massage and acupuncture. One higher quality trial showed a large positive effect of 12-month use of a night splint on hand pain, function, strength and range of motion. Exercise had no effect on hand pain or function although it may be able to improve hand strength. Low level laser therapy may be useful for improving range of motion. No rehabilitation interventions were found to improve stiffness.

**Conclusions:**

There is emerging high quality evidence to support that rehabilitation interventions can offer significant benefits to individuals with hand OA. A summary of the higher quality evidence is provided to assist with clinical decision making based on current evidence. Further high-quality research is needed concerning the effects of rehabilitation interventions on specific treatment goals for hand OA.

## Introduction

Hand osteoarthritis (OA) is a common chronic condition involving one or more joints of the thumb and fingers [1]. Estimates of the prevalence of symptomatic hand OA range from 13% to 26% and are greater in women [1]. Hand OA is associated with pain, reduced grip strength, loss of range of motion (ROM), and joint stiffness, leading to impaired hand function and difficulty with daily activities [2].

According to the European League Against Rheumatism (EULAR), the optimal management of hand OA requires both non-pharmacological and pharmacological approaches [1]. Rehabilitative interventions are both non-pharmacological and non-surgical treatments used by therapists in clinical practice to help maintain or regain a person's maximum self-sufficiency and function. They include treatments such as exercise, splints, heat therapy, electrotherapy, acupuncture, and massage and are recommended for relieving pain and improving hand function, although the level of evidence supporting this recommendation is mainly at the level of 'expert opinion' [1].

Common goals for the treatment of hand OA are pain relief, improved hand strength and ROM, and reduced stiffness, with an overall goal to improve physical hand function [3]. Evidence-based practice requires knowledge of which interventions will most effectively address treatment goals and which interventions best target prioritized problems [4].

To date, there have been five systematic reviews [5-9] investigating conservative interventions for hand OA. The focus of the two earliest reviews was on pharmacological interventions, with little emphasis given to rehabilitative treatments [6,9]. Although Towheed's systematic review [8] and its update [5] reviewed studies of rehabilitative approaches, the main emphasis of these reviews was on methodological quality rather than treatment effects. The effectiveness of different rehabilitation interventions on specific treatment goals has not yet been fully explored. The most recently published systematic review [7] summarized the evidence based on systematic reviews rather than relevant primary studies. Its most striking finding was the paucity of available systematic reviews in this area and limited quality evidence that can be used to guide best practice.

Given the prevalence of hand OA and the limited evidence for non-pharmacological conservative treatments, the objectives of this systematic review were (a) to review the current quality of evidence of rehabilitation interventions for hand OA; (b) to explore the treatment effects of these rehabilitation treatments in relation to specific outcome measures of hand pain, strength, ROM, and stiffness and to hand function in adults with hand OA; and (c) to provide evidence-based knowledge on the treatment effects of different rehabilitation interventions for specific treatment goals.

Knowledge of study quality and the treatment effects of specific rehabilitation techniques will be useful to help guide best clinical practice for individuals with a diagnosis of hand OA. Greater knowledge of which treatments offer the greatest effect on specific treatment goals will aid therapists to select the most effective rehabilitation strategies to improve impairment and function in individuals with hand OA. Evidence of treatment effects from higher-quality studies can be used in clinical practice to guide informed decision making and meet patient-specific goals.

## Materials and methods

### Eligibility criteria

Randomized controlled trials (RCTs), quasi-RCTs, or crossover trials (that is, level of evidence 1b and 2b on Oxford levels of evidence) [10] in English were included for evaluation if they compared some form of rehabilitation with a control for adults whose condition was diagnosed as hand OA. The rehabilitative interventions included those that are used by therapists in clinical practice to treat hand OA, such as exercise, splints, heat therapy, electrotherapy, acupuncture, and massage. The control could be no treatment, usual care, or a placebo intervention. In addition, studies needed to assess at least one of the following outcomes: (a) hand pain including individual joint(s) or overall hand pain, (b) self-reported hand physical function, or (c) other measures of hand impairment, such as grip strength, ROM, or stiffness. Studies evaluating surgical or pharmacological interventions were excluded as were studies reported only in the form of abstracts, conference proceedings, or poster presentations.

### Search strategy

We searched the following electronic databases: MEDLINE (1950 to October 2010), CINAHL (Cumulative Index to Nursing and Allied Health Literature) (1981 to October 2010), ISI Web of Science (1950 to October 2010), SciVerse Scopus (1960 to October 2010), and Physiotherapy Evidence Database (PEDro) (1999). Specific search strategies for each database are provided in Appendix 1 (Additional file 1). We also searched the references of all systematic reviews of hand OA [5-9] and papers from experts in the field.

### Study selection

We examined the list of titles and abstracts identified by the literature searches for potentially relevant studies. Two reviewers (LY and LK) independently applied the predetermined inclusion criteria to the full text of the identified studies. Any conflicts were resolved through a third independent researcher (KB).

### Assessment of study quality

Two independent raters (LY and LK) assessed the methodological quality of included trials by means of the PEDro scale [11]. Disagreements were resolved by discussion with a third reviewer (KB). The PEDro scale is a validated scale used to assess the quality of randomized controlled rehabilitative studies [12-14] and provides a comprehensive measure of methodological quality [15]. It includes 11 criteria to assess the internal and external validity of clinical trials: criterion 1 measures external validity and is not included in the final score, and criteria 2 to 11 measure internal validity. The scale is scored out of 10, with 10 indicating the highest quality and 0 indicating the poorest quality. The items consist of (1) specification of eligibility criteria, (2) random allocation, (3) concealed allocation, (4) similarity at baseline, (5) blinding of subjects, (6) blinding of operators, (7) blinding of assessors, (8) measures of at least one key outcome obtained from at least 85% of subjects initially allocated to groups, (9) intention-to-treat principle, (10) results of between-group comparison, and (11) point measures and measures of variability reported. As it is difficult to blind therapists or participants in most rehabilitation trials, many studies do not meet all criteria; therefore, a trial can be considered to be of relatively high quality if it scores greater than 6 out of 10 on the PEDro scale [16].

### Date extraction and analysis

A predefined data extraction form with study design, participant characteristics, diagnosis, affected hand joints, intervention, and duration of interventions was used. To provide a comparison between outcomes reported by the studies, the standardized mean difference (SMD) over time and corresponding 95% confidence interval (CI) were calculated for continuous variables, if possible, immediately after treatment and at the longest follow-up time point by means of the software package RevMan 5 [17]. Although studies may have provided more than one outcome measure under each category of pain, function, strength, ROM, and stiffness, only one measure in each category per study was selected. The measures selected for calculation of the SMD were based on the following hierarchy: (a) for pain, measures of global hand pain took precedence over pain on motion and the Australian/Canadian OA hand index (AUSCAN) pain subscale [18]; (b) for strength, grip strength took precedence over lateral pinch strength and other strength as grip strength is the most commonly used outcome measure in these trials; and (c) for trials measuring outcomes for different hand joints, we extracted data of the joints in the following order: the distal interphalangeal (DIP) joints, the base of the thumb carpometacarpal (CMC) joints, and the proximal interphalangeal (PIP) joints, as the most commonly affected hand joints, in decreasing order, are the DIP joints, thumb CMC joints, and the PIP joints [19]. The effect estimates were interpreted as described by Cohen [20]; that is, an SMD of 0.2 to 0.5 was considered a small effect, 0.5 to 0.8 a moderate effect, and at least 0.8 a large effect of the individual rehabilitative intervention. We had planned to conduct a meta-analysis but this was not possible, owing to the heterogeneity of study interventions and outcome measures, which made pooling of data across trials inappropriate (I^2 ^values of 89% to 99%).

## Results

### Study selection

**Figure 1:**
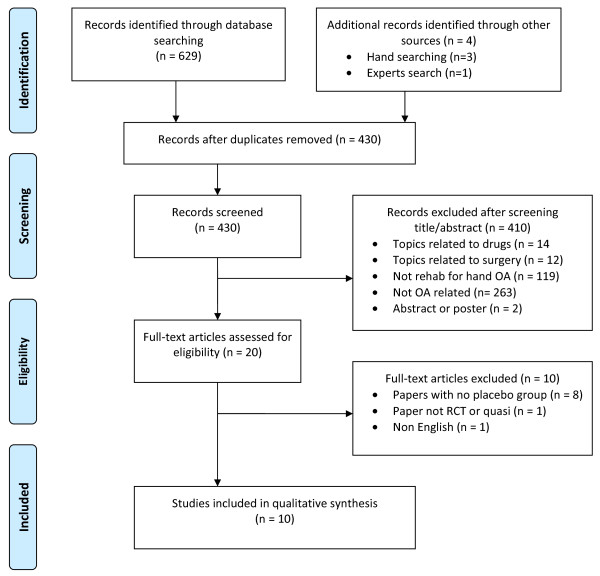
**Flow diagram of the results of the study selection procedure, which is in accordance with Preferred Reporting Items for Systematic Reviews and Meta-Analyses (PRISMA) guidelines**. OA, osteoarthritis; RCT, randomized controlled trial.

A flow diagram, in accordance with the Preferred Reporting Items for Systematic Reviews and Meta-Analyses (PRISMA) guidelines [21], of the results of the study selection procedure is presented in Figure 1. The search strategy yielded 629 articles. After duplications were deleted, 430 articles remained. Of these, 20 studies met the inclusion criteria [22-41]. After the full-text versions of these papers were reviewed, 10 studies were selected for this systematic review [22,24,26,27,30,31,33-35,39]. Reasons for exclusion included lack of a control group (*n *= 8) [23,25,32,36-38,40,41], language other than English (*n *= 1) [28], and not RCT or quasi-RCT (*n *= 1) [29].

### Study characteristics

Details of the 10 eligible studies are presented in Tables 1 and 2. Of these studies, seven were RCTs, two were crossover trials, and one was a quasi-RCT. Five studies involved patients with both CMC joint and interphalangeal (IP) joint OA, one study involved patients with OA of the CMC joint only, while the remainder did not report the specific hand joints involved. Diagnosis of hand OA was based on clinical or radiologic criteria (or both) in five studies and on clinical criteria only in three studies; two studies did not clearly state their method of diagnosing hand OA. The age of participants ranged from 56 to 82 years, which is representative of adults with OA as reported in cohort studies [42,43]. Six different rehabilitation interventions were investigated (Table 2): one study investigated splints [31], two investigated laser therapy [22,24], two investigated heat therapy (using infrared radiation from a lamp or a heated tiled stove) [35,39], three investigated exercise programs [30,33,34], one investigated massage [27], and one investigated acupuncture [26]. Treatment durations ranged from 2 to 52 weeks, with a mean (standard deviation) of 10.9 (15.1) weeks. All studies, except one [39], reported the outcome measures immediately after treatment. Two studies reported a longer-term follow-up, with durations ranging from 2 weeks to 1 year [24,31].

**Table 1:** Study design and participant characteristics

Reference	Study design	LOE	Total, n	Gender		Age, years		Diagnosis of hand OA		CMC joint OA	IP joint OA
								
				M, n	F, n	Mean (SD)					
						Intervention	Control	Clinical	Radiology		
Rannou, *et al*. [31]	RCT	1b	112	11	101	63 (8)	64 (8)	Yes	Yes	Yes	Yes
Basford, *et al*. [22]	RCT	1b	81	NS	NS	57 (NS)	63 (NS)	Yes	No	Yes	Yes
Brosseau, *et al*. [24]	RCT	1b	88	19	69	64 (10)	65 (10)	Yes	Yes	Yes	Yes
Stange-Rezende, *et al*. [35]	Crossover	2b	45	3	42	60 (8)	60 (8)	Yes	No	Yes	Yes
Favaro, *et al*. [39]	Quasi-RCT	2b	48	5	43	56 (6)	60 (8)	Yes	Yes	NS	NS
Stamm, *et al*. [34]	RCT	2b	40	5	35	61 (8)	60 (8)	Yes	No	Yes	Yes
Lefler and Armstrong [30]	RCT	2b Yes	19	2	17	82 (10)	82 (8)	NS	NS	NS	NS
Rogers and Wilder [33]	Crossover	2b	76	11	65	75 (7)	75 (7)	Yes	Yes	NS	NS
Field, *et al*. [27]	RCT	2b Yes	22	1	21	NS	NS	NS	NS	NS	NS
Dickens and Lewith [26]	RCT	1b	13	5	7	59 (9)	59.2 (6)	Yes	Yes	Yes	No

CMC, carpometacarpal; F, female; IP, interphalageal; LOE, level of evidence (Oxford); M, male; n, number; NS, not stated; OA, osteoarthritis; RCT, randomized controlled trial; SD, standard deviation.

**Table 2:** Description of study interventions and outcome measures

Study	Intervention	Control intervention	Intervention duration	Post-treatment measurements	Outcome measures
Rannou, *et al*. [31]	Use of splint at night only	Usual care based on physician's discretion	1 year	1 month (use of splint) Immediate	VAS (previous 48 hours) VAS during pinch CHFS Pinch strength Kapandji index
Basford, *et al*. [22]	Laser (15 seconds × 4 points) × 3 sessions/week	Sham laser (15 seconds × 4 points) × 3 sessions/week	3 weeks	Immediate	Joint tenderness of thumb CMC, MCP, and IP and of other joints (0-5) Grasp, lateral pinch, and 3-finger chuck pinch strength Thumb CMC planar and palmar abduction, thumb MCP extension and flexion, and thumb IP extension and flexion
Brosseau, *et al*. [24]	Laser (1 second × 74 points) × 20 minutes/session × 3 sessions/week	Sham laser (1 second × 74 points) × 20 minutes/session × 3 sessions/week	6 weeks	Immediate 6 weeks 12 weeks 24 weeks	AUSCAN VAS (data not available) Lateral pinch and 3-finger chuck pinch strength CMC flexion and opposition, DIP flexion, MCP flexion, and PIP flexion ROM
Stange-Rezende, *et al*. [35]	Room with heated tiled stove (≥3 hours × 3 sessions/week) + customary treatment (as for control)	Customary treatment (NSAIDs, analgesics, home exercises, physiotherapy)	3 weeks	Immediate	VAS (general pain; in hands and global hand function) AUSCAN Grip strength
Favaro, *et al*. [39]	Infrared radiation (20 minutes/sessions × 10 sessions)	Sham infrared radiation (not reported)	Not reported	Not reported	Grip strength
Stamm, *et al*. [34]	Joint protection program - written instructions plus home exercise program (7 ROM exercises × 10 times daily)	Education about OA (20-minute session) plus use of non-slip matting to open jars	3 months	Immediate	Self-reported global hand function - HAQ Grip strength
Lefler and Armstrong [30]	Strengthening exercise program × 3 sessions/week	No treatment	6 weeks	Immediate	Pain (0-6) Grip, palmar, 2nd-5th digit, and lateral pinch strength Finger joint ROM
Rogers and Wilder [33]	Exercise program (6 ROM exercises and 3 strengthening exercises) (10 to 15 minutes daily)	Sham hand cream (cream was applied once daily using gentle technique)	16 weeks	Immediate	AUSCAN Maximal right grip strength and other grip and pinch strength
Field, *et al*. [27]	Massage on wrist/hand (once/week) + daily home self-massage	No treatment	4 weeks	Immediate	VAS anchored with 5 faces (VITAS) Perceived grip strength
Dickens and Lewith [26]	Acupuncture (6 sessions over 2 weeks)	Mock transcutaneous electrical nerve stimulation (6 sessions over 2 weeks)	2 weeks	Immediate 2 weeks	VAS in general, joint tenderness Functional score Pinch strength

ROM refers to active range of motion of carpometacarpal (CMC), metacarpophalangeal (MCP), and interphalangeal (IP) of the thumb and MCP, distal interphalangeal (DIP), and proximal interphalangeal (PIP) joint movements of the 2nd-5th fingers. AUSCAN, Australian/Canadian osteoarthritis hand index; CHFS, Cochin hand functional scale; HAQ, Health Assessment Questionnaire; NSAID, nonsteroidal anti-inflammatory drug; OA, osteoarthritis; VAS, visual analogue scale.

### Methodological quality

The methodological quality of included studies (Table 3) ranged from 3 to 10 points out of a maximum of 10 points. Six trials were considered to have relatively high quality [22,24,26,31,34,35] and four trials lower quality [27,30,33,39]. One study, investigating laser therapy [24], met the criteria of blinding therapists and participants. Concealed allocation and the use of an intention-to-treat analysis were other criteria not met in most studies.

**Table 3:** Quality ratings of included studies according to the PEDro methodology scoring system

Study	Random assignment	Concealed allocation	Groups similar at baseline	Subject blind	Therapist blind	Assessor blind	<15% dropout	ITT analysis	Between-group analysis	Point measures	Score on PEDro scale
Rannou, *et al*. [31]	Yes	Yes	Yes	No	No	Yes	Yes	Yes	Yes	Yes	8
Basford, *et al*. [22]	Yes	No	Yes	Yes	No	Yes	Yes	Yes	Yes	Yes	8
Brosseau, *et al*. [24]	Yes	Yes	Yes	Yes	Yes	Yes	Yes	Yes	Yes	Yes	10
Stange-Rezende, *et al*. [35]	Yes	No	Yes	No	No	Yes	No	Yes	Yes	Yes	6
Favaro, *et al*. [39]	No	No	Yes	Yes	No	No	Yes	No	Yes	Yes	5
Stamm, *et al*. [34]	Yes	No	Yes	No	No	Yes	Yes	No	Yes	Yes	6
Lefler and Armstrong [30]	Yes	No	Yes	No	No	No	Yes	No	Yes	Yes	5
Rogers and Wilder [33]	Yes	No	Yes	Yes	No	No	No	No	Yes	No	4
Field, *et al*. [27]	Yes	No	No	No	No	No	No	No	Yes	Yes	3
Dickens and Lewith [26]	Yes	Yes	No	No	No	Yes	Yes	No	Yes	Yes	6

ITT, intention-to-treat; PEDro, Physiotherapy Evidence Database.

### Results of studies

**Figure 2:**
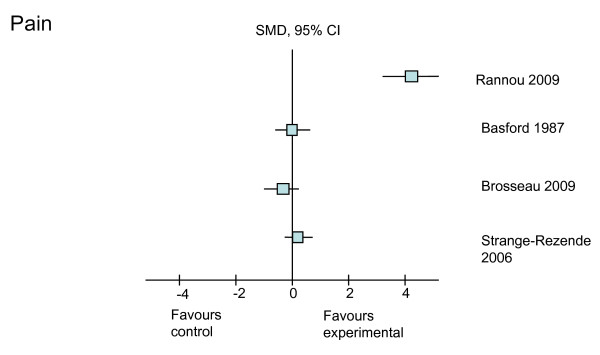
**Treatment effects of the higher-quality studies on pain**. CI, confidence interval; SMD, standardized mean difference.

**Figure 3:**
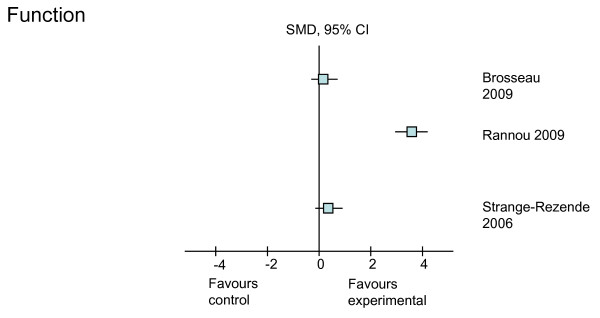
**Treatment effects of the higher-quality studies on function**. CI, confidence interval; SMD, standardized mean difference.

**Figure 4:**
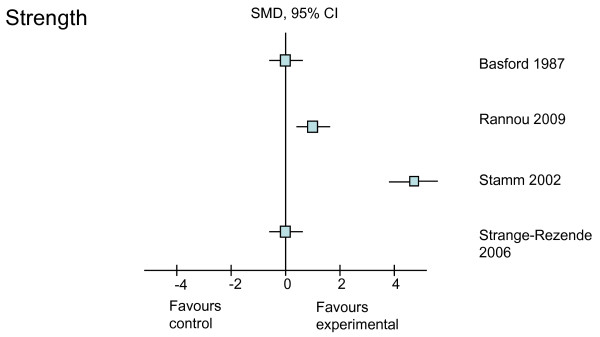
**Treatment effects of the higher-quality studies on strength**. CI, confidence interval; SMD, standardized mean difference.

**Figure 5:**
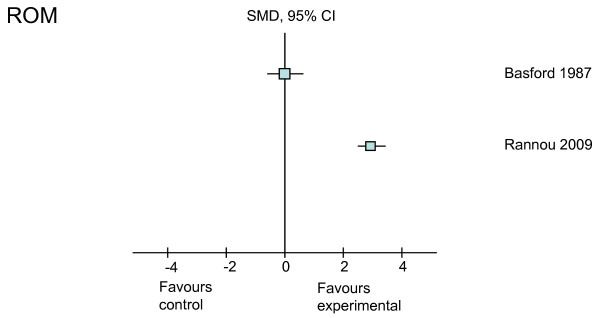
**Treatment effects of the higher-quality studies on range of motion (ROM)**. CI, confidence interval; SMD, standardized mean difference.

**Figure 6:**
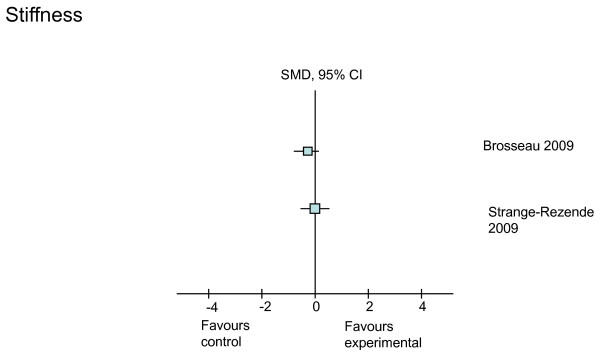
**Treatment effects of the higher-quality studies on stiffness**. CI, confidence interval; SMD, standardized mean difference.

The treatment effects (SMD with 95% CI) of the six different rehabilitative interventions on the outcomes of pain, self-reported physical function, strength, ROM, and self-reported stiffness, immediately after treatment and at the longest follow-up time point, are presented in Table 4. Treatment effects from the higher-quality studies on each of the outcomes are shown in Figures 2, 3, 4, 5 and 6. Most studies focused on interventions to improve pain and strength. Fewer studies investigated the effects on improving function, which is an important goal in clinical practice. Seven studies reported sufficient data to calculate the SMD with its 95% CI. For the remaining three studies, the author or authors were contacted, resulting in additional information from which to calculate the SMD in one of these three studies. The following sections will outline the treatment effects of rehabilitation strategies for each of the included outcomes.

**Table 4:** Treatment effects of rehabilitation interventions on study outcomes

Outcome	Intervention	Study	Measurement tool	Number	SMD (95% CI)	Quality: score on PEDro scale
Pain	Splints	Rannou, *et al*. [31]	VAS	101	0.19 (-0.20, 0.58)	8
	Long-term			97	4.24^a ^(3.52, 4.97)	
	Laser	Basford, *et al*. [22]	0-5 tenderness	81	0.00 (-0.44, 0.44)	8
		Brosseau, *et al*. [24]	AUSCAN pain	86	0.33 (-0.10, 0.75)	10
	Long-term				-0.88 (-0.5, 0.35)	
	Heat therapy	Stange-Rezende, *et al*. [35]	VAS	45	0.09 (-0.32, 0.05)	6
	Exercise	Lefler and Armstrong [30]	0-6 pain scale	18	0.40 (-0.56, 1.36)	5
		Rogers and Wilder [33]	AUSCAN pain	46	-0.04 (-0.45, 0.37)	4
	Massage	Field, *et al*. [27]	VITAS	22	1.18^a ^(0.26, 2.10)	3
	Acupuncture	Dickens and Lewith [26]	VAS	13	NA	6
Hand function	Splints	Rannou, *et al*. [31]	CHFS	101	1.10^a ^(0.68, 1.52)	8
	Long-term			95	3.73^a ^(3.05,4.40)	
	Laser	Brosseau, *et al*. [24]	AUSCAN ADL	86	0.08 (-0.34, 0.50)	10
	Long-term				-0.05 (-0.48, 0.37	
	Heat therapy	Stange-Rezende, *et al*. [35]	AUSCAN	45	0.20 (-0.27, 0.67)	6
	Exercise	Stamm, *et al*. [34]	HAQ	40	NA	6
		Rogers and Wilder [33]	AUSCAN ADL	35	-0.08 (-0.55,0.39)	4
	Acupuncture	Dickens and Lewith [26]	NS	13	NA	6
Hand strength	Splints	Rannou, *et al*. [31]	Pinch (Dy)	96	0.9^a ^(0.5, 1.3)	8
	Long-term				1.2^a ^(0.8, 1.6)	
	Laser	Basford, *et al*. [22]	Grasp (Dy)	81	0.01 (-0.4, 0.5)	8
		Brosseau, *et al*. [24]	Grip (Dy)	86	NA	10
	Heat therapy	Stange-Rezende, *et al*. [35]	Grip (NS)	45	0.00 (-0.4, 0.4)	6
		Favaro, *et al*. [39]	Grip (S)	48	NA	5
	Exercise	Stamm, *et al*. [34]	Grip (V)	40	4.5^a ^(3.3, 5.7)	6
		Lefler and Armstrong [30]	Grip (Dy)	18	0.7 (-0.13, 1.7)	5
		Rogers and Wilder [33]	Grip (Dy)	31	0.2 (-0.3, 0.7)	4
	Massage	Field, *et al*. [27]	Grip -10-point scale	22	0.9 (-0.01, 1.7)	3
	Acupuncture	Dickens and Lewith [26]	Pinch (NS)	13	NA	6
Range of motion	Splints	Rannou, *et al*. [31]	KI	97	-0.4^a ^(-0.8, -0.03)	8
	Long-term				3.30^a ^(2.7, 3.9)	
	Laser	Basford, *et al*. [22]	Goniometer	81	0.00 (-0.4, 0.5)	8
		Brosseau, *et al*. [24]	Goniometer	86	NA	10
	Exercise	Lefler and Armstrong [30]	Goniometer	18	NA	5
Stiffness	Laser	Brosseau, *et al*. [24]	AUSCAN stiffness	86	0.30 (-0.1, 0.7)	10
	Long-term				-0.4 (-0.8, 0)	
	Heat therapy	Stange-Rezende, *et al*. [35]	AUSCAN stiffness	45	-0.04 (-0.3, 0.2)	6
	Exercise	Rogers and Wilder [33]	AUSCAN stiffness	31	3.00 (-45, 51)	4

^a^Significant treatment effects. ADL, activities of daily living; AUSCAN, Australian/Canadian osteoarthritis hand index; CHFS, Cochin hand functional scale; CI, confidence intervals for continuous variables; Dy, dynamometer(s); HAQ, Health Assessment Questionnaire; KI, Kapandji index (thumb opposition); NA, standardized mean difference not estimable; NS, measurement tool not stated; PEDro, Physiotherapy Evidence Database; S, sphygmomanometer; SMD, standardized mean difference; V, vigorimeter; VAS, visual analogue scale; VITAS, visual analogue scale anchored with five faces.

### Pain

The effects of all six rehabilitation interventions on pain were reported in eight of the 10 studies (Table 4). From the eight studies, six were graded as higher quality (greater than 6 on the PEDro scale). Of these higher-quality studies, only one study investigating long-term splint use was shown to have a positive treatment effect on improving pain when the visual analogue scale was used to measure outcome (Figure 2). In this study, Rannou and colleagues [31] found that 12 months of continued use of a night splint resulted in large improvements in pain (SMD = 4.24, 95% CI 3.52, 4.97). One lower-quality study demonstrated a smaller treatment effect of massage on improving pain (SMD = 1.18, 95% CI 0.26, 2.10) [29]. Although we could not calculate the SMD, the authors of the one trial of acupuncture reported no short-term pain-relieving effects (*P *= 1.0) [26].

### Self-reported hand function

The effects of all interventions, except massage, were investigated on hand function in six of the 10 studies (Table 4). From the six studies, five were graded as higher-quality studies. Of these higher-quality studies, a positive treatment effect could be calculated from one study. In this study [31], use of a splint resulted in a large improvement in hand function in both the short and long term as measured by the Cochin hand functional scale (SMD = 1.10 and 3.73, respectively) (Figure 3). Of the two studies from which we were unable to calculate SMD, a significantly higher proportion of patients reported improved function with a 3-month hand ROM exercise program and education about joint protection in comparison with those who received general OA education and use of non-slip matting to open jars (*P *< 0.05) [34]. However, no functional improvement was shown in another exercise trial that included both ROM and strengthening exercises [33]. Laser therapy [24] and heat treatment [35] had no effect on hand function as measured by the AUSCAN. Similarly, the trial on acupuncture reported no effect on function [26].

### Strength

The effects of all interventions on hand strength were investigated in all 10 trials (Table 4). Six of these 10 studies were graded as higher-quality studies, and positive treatment effects could be calculated from two of the six studies (Figure 4). Improvements in hand strength, measured by means of an electronic dynamometer, were found in both the short and long term with the use of splinting in one study (SMD = 0.9 and 1.2, respectively) [31]. A large positive treatment effect (SMD = 4.5), measured by means of a vigorimeter, was found with the use of a home ROM exercise program [34]. Effect sizes could not be calculated in three studies [24,26,39]. Of these studies, one study [24] reported significant improvement in grip strength (*P *= 0.041) when measured with a dynamometer following laser therapy, one trial [39] did not measure between-group strength difference, and the other trial [26] drew no conclusion on the effect of acupuncture on hand strength.

### Range of motion

The effects of three interventions (splints, laser, and exercise) on ROM were investigated by four studies (Table 4). Of these, three were graded as higher-quality studies, and treatment effects could be calculated from one of the three studies. A small negative effect (SMD = -0.4) in the short term and a large positive effect (SMD = 3.3) in the long term were found on hand ROM in one trial of splinting [31] (Figure 5). Of the two studies from which we were unable to calculate SMD, a significant improvement in ROM was reported for hand-strengthening exercises [30] whereas no overall improvement was reported for laser therapy [22,24], except CMC opposition (*P *= 0.011) [24].

### Stiffness

The effects of three interventions (laser, heat, and exercise) on self-reported stiffness using the AUSCAN scale were investigated in three studies, two of which were graded as higher-quality studies (Table 4). None of the interventions had positive treatment effects on hand joint stiffness (Figure 6). However, as stiffness was measured with only one item from the 15-item AUSCAN scale, it is possible that this tool did not capture the full dimension of stiffness.

### Synthesis of results

A summary of current available evidence from higher-quality studies with positive treatment effects of rehabilitative interventions on pain, function, and physical impairments is provided in Table 5.

**Table 5:** Summary of the higher-quality evidence for treating impairments and function in individuals with hand osteoarthritis

Treatment goals	Joints	Intervention	LOE	Quality: score on PEDro scale	Outcome tool	SMD (95% CI)
Pain reduction	CMC + IP	Splints: long-term night use (>12 months) [31]	1b	8	VAS	4.24 (3.52, 4.97)
Improve hand function	CMC + IP	Splints: Short-term night use (1 month) [31]	1b	8	CHFS	1.10 (0.68, 1.52)
		Splints: Long-term night use (>12 months) [31]				3.73 (3.05,4.40)
	CMC + IP	Joint protection education plus home exercise program [34]	2b	6	HAQ	NA, *P *< 0.05
Improve hand strength	CMC + IP	Splints: Short-term night use (1 month) [31]	1b	8	Pinch (Dy)	0.9 (0.5, 1.3)
		Splints: Long-term night use (>12 months) [31]				1.2 (0.8, 1.6)
	CMC + IP	Joint protection education plus home exercise program [34]	2b	6	Grip (V)	4.5 (3.3, 5.7)
Improve range of motion	CMC + IP	Splints: Long-term night use (>12 months) [31]	1b	8	KI	3.30 (2.7, 3.9)
	CMC	Low-level laser (20 minutes/session × 3 sessions/week) [24]	1b	10	G	NA, *P *= 0.011
Decrease stiffness	-	-	-	-	-	-

CHFS, Cochin hand functional scale; CI, confidence intervals for continuous variables; CMC, carpometacarpal; Dy, dynamometer(s); G, goniometer (s); HAQ, Health Assessment Questionnaire; IP, interphalangeal; KI, Kapandji index (thumb opposition); LOE, level of evidence (Oxford); NA, standardized mean difference not estimable; PEDro, Physiotherapy Evidence Database; SMD, standardized mean difference; V, vigorimeter VAS, visual analogue scale.

## Discussion

This systematic review revealed very few high-quality clinical trials, particularly given the range of rehabilitative interventions that are available to clinicians for the management of hand OA and that are recommended by international bodies. Given the limited amount and varying quality of evidence, firm conclusions about the benefits of various rehabilitation interventions on specific treatment goals cannot be fully drawn from the results of this review. This review does, however, establish that there is emerging high-quality evidence to support the use of common rehabilitation interventions to treat individuals with hand OA. It also suggests which interventions most effectively target specific treatment goals for hand OA.

### Pain relief and function

Pain relief has been reported as the primary treatment goal for hand OA because of its direct correlation with increased hand function [44]. In this review, the use of long-term night splinting was found to be the only effective intervention for both pain reduction and improved physical function [24]. This relative paucity of effect on pain is somewhat surprising given that RCTs for knee and hip OA have reported positive effects on pain from a variety of rehabilitative interventions [45]. However, this discrepancy may reflect the different disease characteristics, such as different risk factors for development and progression, biomechanical features, and physical impairments of hand OA when compared with lower-extremity OA.

Night splinting of the thumb has particularly been recommended for OA of the hand [46] as CMC joint OA has a greater impact on pain and dysfunction than IP OA does [47]. A 7-year prospective study [48] showed that thumb splinting improved hand function and, importantly, reduced the need for surgery. EULAR [49] also recommends using splints to prevent/correct lateral angulation and flexion deformity at the thumb. Our review found evidence from a higher-quality adequately powered RCT that a custom-made neoprene night splint led to significant improvements compared with usual care for 12 months, although it did not improve pain or ROM in the short term (1 month) [31]. In the trial by Rannou and colleagues [31], participants were instructed to use the night splint for 12 months. Adherence was good: 86% wore the splint 5 to 7 nights a week [31].

Evidence from this review did not support the use of laser therapy, heat treatment, exercise, or acupuncture for reducing both pain and improving function in hand OA. However, Stamm and colleagues [34] reported a higher proportion of patients with an at least 10% increase in global hand function using exercise. This was the only exercise study to report an improvement in hand function; however, as the exercise was combined with joint protection education, it is difficult to truly isolate the independent effects of exercise [34].

Low-level laser therapy has been found to regulate chondrocytic proliferation and stimulate collagen synthesis in animals [50,51]. It is thought to have analgesic effects as well as biomodulatory effects of microcirculation [52]. Despite these physiological effects, the two high-quality, well-powered RCTs in our review reported no significant positive clinical effects of laser therapy delivered thrice weekly for 3 to 6 weeks on pain and hand function. This contrasts with findings for laser therapy in the treatment of knee OA, for which there is moderate-quality evidence of beneficial effects, including pain reduction and functional improvement [53,54]. It may be that different devices, method and site of application, wavelength, treatment regime, and measurement tools influence the result.

Massage therapy was shown to be effective in reducing pain in patients with hand OA; however, owing to the lower quality (3 on the PEDro scale) of the one study on massage [27], it is hard to draw definitive conclusions about massage therapy. The single trial of acupuncture did not support its use for hand OA for pain and function, but no detail was provided about the treatment dosage, including the acupuncture points, used. This lack of effect of acupuncture is consistent with findings of a recent systematic review of acupuncture for all OA; the review showed that, while there were statistically significant benefits in sham-controlled trials, the benefits were small, did not meet predefined thresholds for clinical relevance, and were possibly due at least partially to placebo effects from incomplete blinding [55].

### Strength, range of motion, and stiffness

Improvements of hand strength and ROM and reduction of stiffness are also common goals of rehabilitation on hand OA [3]. The use of night splints in both the short term and long term was shown to have a treatment effect on strength and ROM but not on stiffness. Interestingly, the use of night splinting produced a small negative treatment effect (SMD = -0.4) in the short term but a large positive effect (SMD = 3.3) in the long term on ROM in one study [24]. This finding is important knowledge for therapists when providing advice on the duration of night splint use when the goal is to improve ROM.

Exercise is considered a mainstay of treatment for OA and yet, in this review, only three RCTs [30,33,34] of lower quality investigated the effects of various exercise programs to improve strength, ROM, or stiffness. Surprisingly, the exercise programs that incorporated strengthening exercises failed to find strength gains yet found an effect on ROM [30,33], while a large significant improvement in grip strength was found with a program that involved ROM exercises [34]. These programs all differed in their exercise content and dosage. Precise details on the intensity of the exercise program were limited. It is possible that the intensity of the strengthening exercises was insufficient for change to occur, especially given that increases in strength were not evident. Further studies that address the optimal intensity of strengthening exercises for hand OA are required.

No studies found significant positive effects of splints, laser, heat, or exercise on stiffness. Further trials using larger sample sizes and a more rigorous methodology are needed to evaluate different forms of exercise on improving strength and ROM and reducing stiffness in patients with hand OA. Constraining outcome measures to only self-reported methods, such as using the 1-item AUSCAN stiffness subscale to measure stiffness, may reduce the ability to capture the full dimension of the impairment [56]. The additional use of performance-based outcome measures that can complement self-reported measures needs to be considered when assessing outcomes, such as stiffness, to assist in capturing this extent of impairment and function in hand OA.

The only other rehabilitation intervention reported to improve strength or ROM was laser therapy [24]. This high-quality, well-powered RCT found a benefit of laser therapy delivered thrice weekly for 3 to 6 weeks on grip strength and CMC opposition. Other treatment modalities investigating the effect of heat therapy for patients with hand OA did not find improvements in strength or stiffness when using either the heat provided by a tiled stove [35] or infrared radiation [39]. No studies on the application of wax or hot packs were included in this review.

### Other treatment modalities

No studies fulfilling our inclusion criteria were found for ultrasound or transcutaneous electrical nerve stimulation (TENS). Ultrasound is recommended by EULAR for the management of OA, yet there is evidence from studies of knee OA that ultrasound offers no benefit over placebo [53]. Given that hand joints are more superficial than the knee joint, ultrasound may have different effects in hand OA and is worthy of investigation. Likewise, the effect of TENS for the management of hand OA should be investigated given that some [53,54] but not all [57] systematic reviews in knee OA show that TENS has significant pain-relieving benefits. One study involving TENS, excluded from our review but included in that of Towheed [8], found that use of a glove electrode was, overall, more effective than use of a carbon electrode when using TENS in individuals with hand OA. Other rehabilitative interventions we excluded from our review involved a yoga program [29], which was reported to be effective in improving pain, tenderness, and ROM, and leech therapy, which was more effective than treatment with the drug diclofenac [58].

There are several limitations to this review. First, the statistical power of most studies was rather low. To detect a medium effect size of 0.5 (with α = 0.5 and power at 80%), the sample size per group needs to be at least 50 [20]. This is particularly relevant given that many studies reported a lack of treatment effect on the measured outcomes, and this lack of effect may simply reflect inadequate statistical power. Furthermore, despite contacting authors requesting additional information where required, we were unable to calculate effect sizes for two trials included in the review. Second, we did not confine our studies to RCTs, given the likely lack of studies in this area, and instead included one quasi-RCT [39] and two crossover trials [33,35] on the assumption that hand OA is a non-curable condition and that carry-over of treatment effect across periods may be less likely. The findings of these studies need to be interpreted cautiously given these study designs. Third, the methodological assessment revealed some threats to the validity of the included trials, with around half the studies rated as being of lower quality. A summary of the evidence was therefore made with higher-quality studies graded by means of the PEDro system. Fourth, there was variable use of outcome measures across the trials, making it difficult to compare and pool results across studies.

## Conclusions

This systematic review establishes that there is emerging high-quality evidence to support that certain rehabilitation interventions provide benefits to specific treatment goals in individuals with hand OA. A summary of the higher-quality evidence is provided to assist with clinical decision making based on current evidence. In this review, the evidence suggests the following: (a) long-term use of a night splint offers significant benefits to improve pain, hand function, strength, and ROM for patients with OA; (b) programs of joint protection, advice, and home exercises are effective at improving grip strength and hand function; (c) low-level laser therapy is effective at improving ROM; and (d) no rehabilitation interventions were found to improve stiffness.

Though recommended for OA, exercise programs have not yet been shown to reduce pain in this patient group. We concur with previous systematic reviews suggesting that further high-quality research is urgently needed concerning the effects of rehabilitation interventions on specific patient goals for individuals with hand OA. Specifically, the future agenda should include (a) the use of a common set of outcome measures that adequately capture the dimensions of impairments and function; (b) the use of higher-quality, well-powered studies that adhere to the CONSORT (Consolidated Standards of Reporting Trials) guidelines for non-pharmacological treatments [59]; and (c) the role of exercise on specific patient goals for individuals with hand OA with consideration of the optimal frequency and intensity of training.

## Abbreviations

AUSCAN: Australian/Canadian osteoarthritis hand index; CI: confidence interval; CMC: carpometacarpal; DIP: distal interphalangeal; EULAR: European League Against Rheumatism; IP: interphalangeal; OA: osteoarthritis; PEDro: Physiotherapy Evidence Database; PIP: proximal interphalangeal; RCT: randomized controlled trial; ROM: range of motion; SMD: standardized mean difference; TENS: transcutaneous electrical nerve stimulation.

## Competing interests

The authors declare that they have no competing interests.

## Authors' contributions

LY participated in the study design and in the acquisition, analysis, and interpretation of data and drafted the manuscript. LK participated in the study design and in the acquisition and analysis of the data and helped to draft the manuscript. AS participated in the study design and in the analysis and interpretation of the data and helped to draft the manuscript. FD participated in data acquisition, analysis, and interpretation and drafted the final revisions of the manuscript. KB participated in the study concept and design and in the interpretation of the data and assisted with the drafting of the manuscript. All authors read and approved the final manuscript.

## Supplementary Material

Additional file 1 **Appendix 1: Detailed search strategy is attached as an appendix**. Click here for file
