# Supplementary material for: Effects of rehabilitative interventions on pain, function and physical impairments in people with hand osteoarthritis: a systematic review
Source: Arthritis Res Ther. 2011 Feb 18;13(1):R28. doi: 10.1186/ar3254 (PMC3241372; doi:10.1186/ar3254)
Supplement: Additional file 1 — Appendix 1: Detailed search strategy is attached as an appendix. [file ar3254-S1.DOCX]

**Appendix: search strategy**

Key words and Medical Subject Headings (MeSH) for MEDLINE and Cumulative Index of Nursing and Allied Health (CINAHL)

| Key words | MEDLINE MeSH | CINAHL MeSH |
| --- | --- | --- |
| Hand | Finger joint  Hand  Hand joints | Hand joints |
| Metacarpophalangeal | Metacarpophalangeal | Metacarpophalangeal joint |
| Interphalangeal | Interphalangeal | Nil |
| Carpometacarpal | Carpometacarpal | Nil |
| Thumb | Thumb | Thumb |
| Osteoarthritis | Osteoarthritis | Osteoarthritis |
| Intervention* | Nil | Nil |
| Rehabilitation | Rehabilitation  /rehabilitation | Rehabilitation |
| Physiotherapy | Physical therapy modalities | Physical therapy |
| Physical therapy | Physical therapy modalities | Physical therapy |
| Occupational therapy | Occupational therapy | Occupational therapy |
| Hand therapy | Rehabilitation | Hand therapy |
| Exercise* | Exercise therapy  Exercise | Exercise |
| Splint* | Splints | Splints |
| Electrotherapy | Electric stimulation therapy | Electrotherapy |
| TENS | Transcutaneous electric nerve stimulation | Transcutaneous electric nerve stimulation |
| Laser* | Lasers  Laser therapy | Lasers |
| Shortwave | Short-wave therapy | Nil |
| Therapeutic ultrasound | Ultrasonic therapy | Ni |
| Infrared | Infrared rays | Infrared therapy |
| Massage* | Massage | Massage  Deep tissue massage |
| Acupuncture | Acupuncture  Acupuncture therapy | Acupuncture |
| Paraffin wax | Nil | Nil |
| Paraffin bath | Nil | Nil |
| Bath | baths | Nil |
| Brace* | Braces | Orthoses |
| Orthosis | Orthortic devices | Orthoses |
| Orthotic | Orthortic devices | Orthoses |
| Heat | Heating | Heat-cold application |
| Hot$pack | Nil | Nil |
| Whirlpool hot$bath | Nil | Nil |
| Surgical | Surgical procedure, operative | Nil |
| Surgery | Surgical procedure, operative  General surgery  /surgery | Surgery |
| Operation | Surgical procedures, operative  /surgery | Nil |

**MEDLINE**

1. “finger joint” in Topic – Add MeSH
2. Hand in Topic – Add MeSH
3. “hand joints” in Topic – Add MeSH
4. Metacarpophalangeal in Topic – Add MeSH
5. Interphalangeal in Topic – Add MeSH
6. Carpometacarpal in Topic – Add MeSH
7. Thumb in Topic – Add MeSH
8. 1 or 2 or 3 or 4 or 5 or 6 or 7
9. Osteoarthritis in Topic – Add MeSH
10. 8 AND 9
11. Rehabilitation in Topic – Add MeSH
12. “physiotherapy” in Topic – Add MeSH
13. “physical therapy” in Topic – Add MeSH
14. “occupational therapy” in Topic – Add MeSH
15. “hand therapy” in Topic – Add MeSH
16. Exercise* in Topic – Add MeSH
17. Splint* in Topic – Add MeSH
18. Electrotherapy in Topic – Add MeSH
19. TENS in Topic – Add MeSH
20. Laser* in Topic – Add MeSH
21. Massage* in Topic – Add MeSH
22. Acupuncture in Topic – Add MeSH
23. Shortwave in Topic – Add MeSH
24. “therapeutic ultrasound” in Topic – Add MeSH
25. Infrared in Topic – Add MeSH
26. Brace* in Topic – Add MeSH
27. Orthosis in Topic – Add MeSH
28. Orthotic in Topic – Add MeSH
29. Bath in Topic – Add MeSH
30. Heat in Topic – Add MeSH
31. 11 or 12 or 13 or 14 or 15 or 16 or 17 or 18 or 19 or 20 or 21 or 22 or 23 or 24 or 25 or 26 or 27 or 28 or 29 or 30
32. 10 AND 31
33. Surgical in Topic – Add MeSH
34. Surgery in Topic – Add MeSH
35. operation in Topic – Add MeSH
36. 33 or 34 or 35
37. 32 NOT 36

**CINAHL**

1. Hand joints in Tx
2. Metacarpophalangeal joint in Tx
3. Thumb in Tx
4. 1 or 2 or 3
5. Osteoarthritis in Tx
6. 4 AND 5
7. Rehabilitation in Tx
8. Physical therapy in Tx
9. Occupational therapy in Tx
10. Hand therapy in Tx
11. Exercise in Tx
12. Electrotherapy in Tx
13. Transcutaneous electric nerve stimulation in Tx
14. Lasers in Tx
15. Infrared in Tx
16. Heat in Tx
17. Massage in Tx
18. Deep tissue massage in Tx
19. Splints in Tx
20. Brace* in Tx
21. Orthosis in Tx
22. Orthotic in Tx
23. Acupuncture in Tx
24. 7 or 8 or 9 or 10 or 11 or 12 or 13 or 14 or 15 or 16 or 17 or 18 or 19 or 20 or 21 or 22 or 23
25. 6 AND 24
26. Surgery in Tx
27. 26 NOT 27

**ISI Web of Science** (keywords)

1. Hand in Topic
2. Metacarpophalangeal in Topic
3. Interphlangeal in Topic
4. “finger joint” in Topic
5. “hand joints’ in Topic
6. Carpometacarpal in Topic
7. Thumb in Topic
8. #1 OR #2 OR #3 OR #4 OR #5 OR #6 OR #7
9. Osteoarthritis in Topic
10. #8 AND #9
11. Rehabilitation in Topic
12. Physiotherapy in Topic
13. “physical therapy” in Topic
14. “occupational therapy” in Topic
15. “hand therapy” in Topic
16. Exercise* in Topic
17. Electrotherapy in Topic
18. TENS in Topic
19. Laser* in Topic
20. Infrared in Topic
21. Heat in Topic
22. Shortwave in Topic
23. Therapeutic ultrasound in Topic
24. Brace* in Topic
25. Orthosis in Topic
26. Orthotic in Topic
27. Massage* in Topic
28. Acupuncture in Topic
29. Splint* in Topic
30. 11 or 12 or 13 or 14 or 15 or 16 or 17 or 18 or 19 or 20 or 21 or 22 or 23 or 24 or 25 or 26 or 27 or 28 or 29
31. 10 AND 30
32. Surgical in Topic
33. Surgery in Topic
34. Operation in Topic
35. 32 or 33 or 34
36. 31 NOT 35

**SCOPUS** (keywords)

1. hand title-abs-key
2. “hand joints” title-abs-key
3. “finger joint” title-abs-key
4. Thumb title-abs-key
5. #1 OR #2 OR #3 OR #4
6. osteoarthritis or arthritis title-abs-key
7. #5 AND #6
8. rehabilitation or “physical therapy” title-abs-key
9. physiotherapy or “occupational therapy” title-abs-key
10. massage* or splint* title-abs-key
11. TENS or laser* title-abs-key
12. “hand therapy” or exercise* title-abs-key
13. Electrotherapy or acupuncture title-abs-key
14. Shortwave or “therapeutic ultrasound” or brace* or “paraffin wax” or “paraffin bath” or orthosis or heat or infrared or “whirlpool hot bath” or “hot pack” title-abs-key
15. #8 OR #9 OR #10 OR #11 OR #12 OR #13 OR #14
16. #7 AND #15
17. Surgical or surgery title-abs-key
18. Operation title-abs-key
19. #17 OR #18
20. #15 AND NOT #19

**PEDro**

1. Hand osteoarthritis
